# Reply to Comments on Paper Published in IJPH as “Factors Associated with Physical Activity among Macedonian Adolescents ...”

**Published:** 2017-02

**Authors:** Seryozha GONTAREV

**Affiliations:** Faculty of Physical Education, Sport, and Health, Ss. Cyril and Methodius University, Skopje, Macedonia

## Dear colleagues

First, I would like to thank you for the constructive remarks. I fully agree with you that the clinical significance is more important than the statistical one. However, the primary aim of this paper was to determine the influence of demographic, psychological, social and environmental factors with physical activity among Macedonian adolescents from Albanian ethnic community. The comparative analysis on the level of physical activity was a secondary aim. The conclusion that boys are physically more active than girls was not made only on the basis of the instrument Physical Activity Questionnaire (Elementary School), which actually just differentiates the examinees - who are less and who are more active, and which cannot determine the percentage of the examinees who have recommended physical activity. This paper was a part of a bigger project researching the physical activity in all ethnical communities in Macedonia for which, apart from the above mentioned instrument, we have used few more instruments for assessment of physical activity, such as IPAQ. We also used Actigraph for a smaller number of examinees (80). Both instruments showed that a much higher percent of boys has recommended physical activity, unlike girls. It is our mistake that we have not mentioned these two instruments in the paper. By the way, we have decided for the instrument Physical Activity Questionnaire (Elementary School) since it proved to be the most valid, i.e. it determined the highest correlation, in comparison with the Actigraph.

We have read the book you have indicated and I think that it will be very helpful for my future researches.

